# Impact of HIV minor species and tropism

**DOI:** 10.1186/1742-4690-9-S1-I21

**Published:** 2012-05-25

**Authors:** Karin Metzner

**Affiliations:** 1 University Hospital, Zurich, Switzerland

## 

Resistance against antiretroviral drugs is one challenge when treating HIV infected subjects. Resistance occurs in consequence of mutations in the HIV genome, thus, viral proteins can no longer be inhibited by antiretroviral drugs or, in case of the CCR5 inhibitor Maraviroc, the virus can also escape through coreceptor switch. Drug-resistant viruses (1) can be rapidly selected leading to virological failure, (2) can persist also in the absence of drugs, (3) can be transmitted, and (4) their presence can limit further treatment options. This is all known in the context of drug-resistant viruses representing the majority of the virus population. Here, the focus will be on minority drug-resistant HIV variants: Virus subpopulations not detectable using standard genotype resistance testing based on population sequencing, thus, representing less than 20-25% of the total virus population. New technologies enable the detection and quantification of minority drug-resistant HIV variants to levels far below 1%. However, their impact on antiretroviral therapy is still controversially discussed. This presentation will provide an overview of the current techniques to quantify minority viral variants, the clinical studies investigating the prevalence and impact of minority drug-resistant HIV variants, and the challenges and potential benefits of clinical implementation.

